# Moderate patchiness optimizes heterogeneity, stability, and beta diversity in mesic grassland

**DOI:** 10.1002/ece3.4081

**Published:** 2018-04-20

**Authors:** Devan Allen McGranahan, Torre J. Hovick, Robert Dwayne Elmore, David M. Engle, Samuel D. Fuhlendorf

**Affiliations:** ^1^ School of Natural Resource Sciences‐Range Science Program North Dakota State University Fargo North Dakota; ^2^ Department of Natural Resource Ecology and Management Oklahoma State University Stillwater Oklahoma

**Keywords:** diversity–stability theory, fire–grazing interaction, heterogeneity‐based management, landscape ecology of fire and grazing, pyric herbivory, rangeland biodiversity

## Abstract

Heterogeneous disturbance patterns are fundamental to rangeland conservation and management because heterogeneity creates patchy vegetation, broadens niche availability, increases compositional dissimilarity, and enhances temporal stability of aboveground biomass production. Pyrodiversity is a popular concept for how variability in fire as an ecological disturbance can enhance heterogeneity, but mechanistic understanding of factors that drive heterogeneity is lacking. Mesic grasslands are examples of ecosystems in which pyrodiversity is linked strongly to broad ecological processes such as trophic interactions because grazers are attracted to recently burned areas, creating a unique ecological disturbance referred to as the fire–grazing interaction, or pyric herbivory. But several questions about the application of pyric herbivory remain: What proportion of a grazed landscape must burn, or how many patches are required, to create sufficient spatial heterogeneity and reduce temporal variability? How frequently should patches burn? Does season of fire matter? To bring theory into applied practice, we studied a gradient of grazed tallgrass prairie landscapes created by different sizes, seasons, and frequencies of fire, and used analyses sensitive to nonlinear trends. The greatest spatial heterogeneity and lowest temporal variability in aboveground plant biomass, and greatest plant functional group beta diversity, occurred in landscapes with three to four patches (25%–33% of area burned) and three‐ to four‐year fire return intervals. Beta diversity had a positive association with spatial heterogeneity and negative relationship with temporal variability. Rather than prescribing that these results constitute best management practices, we emphasize the flexibility offered by interactions between patch number and fire frequency for matching rangeland productivity and offtake to specific management goals. As we observed no differences across season of fire, we recommend future research focus on fire frequency within a moderate proportion of the landscape burned, and consider a wider seasonal burn window.

## 1 INTRODUCTION

Motivated by a concern that biodiversity suffers from homogenous vegetation structure, Fuhlendorf et al. (2006) posed a question: “Should heterogeneity be the basis for conservation?” A decade on, the answer appears to be, Yes—across a breadth of taxa, including birds, invertebrates, and small mammals, landscape‐level heterogeneity has been repeatedly shown to increase two ecosystem properties critical to biodiversity conservation: the structural diversity of vegetation among patches, and dissimilarity in community composition across patches, when compared to homogeneous landscapes (Fuhlendorf, Fynn, McGranahan, & Twidwell, 2017). These outcomes are compelling and are often sought by ecosystem managers; thus, it is of little surprise that enhancing heterogeneity has become a common objective for conservation and management (McGranahan & Kirkman, 2013; Beale et al., 2013; Scasta et al., 2016).

But a mechanistic understanding of the processes relating heterogeneity and biodiversity remains elusive, despite substantial research supporting the pattern of heterogeneity enhancing biodiversity. In many ecosystems, heterogeneity is attributed to spatial and temporal variability in disturbance regimes, especially fire, which suggests “pyrodiversity begets biodiversity.” But this paradigm has received critique for want of scientific rigor in evaluating outcomes and making management prescriptions (Parr & Andersen, 2006). One approach to a functional understanding of pyrodiversity is to frame it in the context of trophic interactions to determine how individual components are affected by variability and alteration of the ecosystem's fire regime (Bowman et al., 2016).

Many mesic rangelands—especially grasslands and savannas—are examples of ecosystems in which pyrodiversity is fundamental to trophic structure because consumers respond strongly to the spatial pattern of fire in the landscape (Archibald, Bond, Stock, & Fairbanks, 2005; Fuhlendorf, Engle, Kerby, & Hamilton, 2009). Thus, pyrodiversity drives landscape‐level heterogeneity by creating contrast in vegetation structure among internally homogeneous patches established through the interaction between fire and herbivory; animals select—and avoid—patches in response to landscape‐level differences in contrasting forage quality created by spatially discrete fire (Archibald et al., 2005; Allred, Fuhlendorf, Engle, & Elmore, 2011; McGranahan et al., 2012). Wildlife biodiversity is in turn enhanced by greater niche breadth created by expanded resource availability along a landscape‐scale gradient of vegetation structure (McGranahan et al., 2013). The temporal variability of aboveground plant biomass in these patches declines as contrast among them increases (McGranahan et al., 2016).

European settlement of rangelands worldwide generally reduced heterogeneity, particularly in North America, which contributed to a decline in rangeland biodiversity as livestock producers sought homogeneous grazing distribution and efficient forage use (Fuhlendorf & Engle, 2001; Fuhlendorf et al., 2017). An alternative paradigm is based on spatial and temporal heterogeneity in disturbance regimes to promote a breadth of ecosystem services in addition to forage and animal protein production (Fuhlendorf, Engle, Elmore, Limb, & Bidwell, 2012). Patch burn‐grazing is the application of spatially discrete prescribed fire to establish a fire–grazing interaction with livestock, which creates an ecologically analogous pattern of disturbance in working landscapes (McGranahan, 2014). In practice, large grazing units of a ranch are conceived of as landscapes in which discrete patches are burned each year to create high‐quality forage for grazing livestock (Toombs, Derner, Augustine, Krueger, & Gallagher, 2010). Managers delineate patches as a certain proportion of the landscape based on their desired fire frequency to ensure consistent, annual fire; for example, burning 25% of the landscape each year creates a 4‐year fire return interval in a landscape with four discrete patches. Additional variability can be introduced by burning in different seasons; for example, a six‐patch system with two burns per year creates a 3‐year fire return interval (Fuhlendorf & Engle, 2004).

Despite greater understanding of the role of heterogeneity in biodiversity conservation, several questions related to ecological applications remain. Generally, much of the research on how heterogeneity‐based management benefits biodiversity uses a conventional treatment–control approach: A treatment in which a single component of the fire regime—the spatial distribution of fire—is manipulated in a fixed, replicated manner is compared to a spatially homogenous control. Results of such studies are exposed to the pyrodiversity critique in that they fail to elucidate mechanisms or inform broad prescriptions because the sensitivity of the response to the single‐level manipulation is unknown (Parr & Andersen, 2006; Foster, Sato, Lindenmayer, & Barton, 2016).

Although the critique arises from failure to integrate individual field studies into ecological theory, there are management implications for the knowledge gaps. Ecosystem managers must balance many environmental variables against logistical and societal constraints, and thus require specific information on the appropriate proportion of the landscape to burn, and when and how frequently to burn, to ensure that heterogeneity enhancement is compatible with other objectives such as livestock production and woody vegetation control as well as fuels and wildfire management (Morton, Regen, Engle, Miller, & Harr, 2010; Twidwell et al., 2013).

Additionally, managers need support on how to monitor outcomes of heterogeneity‐based management using conventional data and analyses. Recent work linking spatial heterogeneity and temporal variability—two important and related ecosystem properties—uses relatively complex statistical frameworks (Wang & Loreau, 2014; McGranahan et al., 2016). But these models are actually based on a classic concept in ecology—Whittaker's (1960) alpha/beta/gamma framework—that has also been extended to analyzing vegetation data many ecosystem managers collect and are familiar with. Beta diversity was the original spatial component of diversity, and has since been extended to represent compositional dissimilarity as a groups’ breadth or range in the multivariate space of an ordination, a conventional community analysis (Anderson, Ellingsen, & McArdle, 2006). Given that patch contrast in heterogeneous rangeland is characterized by dissimilarity in plant functional group composition (Fuhlendorf et al., 2006; McGranahan et al., 2013), using multivariate dispersion—the range of spread in ordination space—as a measure of beta diversity might also correlate with spatial heterogeneity (Anderson et al., 2006). Together, describing spatial heterogeneity and temporal variability in terms of contrast among patches for productivity and structure, and beta diversity for composition, might present a useful framework for managers to assess heterogeneity‐based management.

Thus, a new research imperative is to improve our understanding of how to manage and measure heterogeneity for conservation. We use vegetation structure and plant functional group composition data from a series of tallgrass prairie landscapes managed with fire and grazing to address the following research questions: (1) Is there an optimal number of patches at which spatial heterogeneity in aboveground plant biomass and plant functional group beta diversity are high, and temporal variability in aboveground plant biomass low? (2) How do patchiness, fire frequency, and season of fire affect heterogeneity‐based outcomes? (3) Does beta diversity have a positive, linear relationship with spatial heterogeneity and a negative, linear relationship with temporal variability?

In the context of these questions, spatial heterogeneity describes patch contrast, or the degree of difference in aboveground plant biomass/vegetation structure between patches within landscapes (McGranahan et al., 2012), and temporal variability is a measure of constancy in vegetation structure across patches through time (Ives & Carpenter, 2007). We measure spatial heterogeneity and temporal variability as the amount of variance allocated to spatial sampling units and study years, respectively, in a variance partitioning model based on random‐effects regression (Winter et al., 2012; McGranahan et al., 2016). We measure beta diversity as multivariate dispersion, or range of spread in an ordination of compositional and vegetation structure data (Anderson et al., 2006). We use a break point analysis—which, based on piecewise regression, is sensitive to nonlinear relationships—to determine whether specific elements of spatial–temporal fire regime maximize response variables, and we use permutational multivariate tests to determine how compositional dissimilarity varies across spatial–temporal fire regimes.

## 2 METHODS

### 2.1 Study site and experimental design

Our data came from a fire–grazing interaction experiment at the Tallgrass Prairie Preserve in northeastern Oklahoma, USA (36°50′52″N, 96°25′25″W), in which a gradient of patchiness was created by manipulating the spatial extent (patch size), frequency (fire return interval), and seasonality of prescribed fire (Hovick, Elmore, Fuhlendorf, Engle, & Hamilton, 2015; McGranahan et al., 2016). The study was initiated in 2008, when seven landscapes (430–980 ha)—from one to eight patches and one‐ to 4‐year fire return intervals—were either burned in the spring or in both the spring and summer (see Appendix S1 for schematic). Larger landscapes generally had more patches; upon initiation, patches and fires were randomly assigned. Landscapes were moderately stocked with cattle (*Bos taurus*) each season. Our data were collected between 2011 and 2013, during which there was an average of 659 mm (±116 *SE*) annual rainfall. Typical upland tallgrass prairie vegetation included tall grasses—*Andropogon gerardii*,* Schizachyrium scoparium*,* Panicum virgatum*, and *Sorghastrum nutans*—and forbs included *Ambrosia*,* Asclepias*,* Helianthus*, and *Vernonia* spp. Shrubs were mostly *Rubus* spp.

Vegetation data were collected mid‐June. All landscapes were sampled identically. Each landscape had 12 plots, randomly placed by a GIS algorithm at least 250 m apart, within which 17 observations on vegetation structure and plant functional group composition were collected at 2.5 m intervals along two, 20‐m transects oriented north—south and east—west that intersected perpendicularly and centered on their midpoints (Hovick et al., 2015). We recorded total aboveground plant biomass and canopy cover of plant functional groups + bare ground and litter cover. We estimated aboveground plant biomass with a modified Nudd's board (Guthery, Doerr, & Taylor, 1981), which combines vegetation height and density into a single measure of visual obstruction (Harrell & Fuhlendorf, 2002) that correlates with aboveground plant biomass (Vermeire, Ganguli, & Gillen, 2002). Coverage of graminoids, forbs, shrubs, bare ground, and litter was visually estimated along the Daubenmire (1959) cover class index within a 0.5‐m^2^ quadrat. For each response variable, plot‐level data were represented by a mean of the 17 observations.

### 2.2 Data analysis

#### 2.2.1 Random‐effects regression on aboveground plant biomass

We used the variance partitioning method of McGranahan et al. (2016) to determine the amount of variance in aboveground plant biomass attributable to two terms in the beta–gamma variability framework of Wang and Loreau (2014): spatial heterogeneity—variance among plots (beta variability)—and temporal variability—variance among years (gamma variability). Our random‐effects generalized linear regression model using a gamma distribution with function glmer in the R package lme4 (Bates, Mächler, Bolker, & Walker, 2015; R Core Team 2016) allocated variance among spatial (plot) and temporal (year) terms for each experimental landscape with visual obstruction as the response variable. Variance data were fit in piecewise and ordinary least squares regression (see below).

#### 2.2.2 Multivariate dispersion as beta diversity

We measured beta diversity as the breadth of groups in ordination space using the betadisper function in the R package vegan (Oksanen et al., 2013). Specifically, we used the mean distance of site scores to group centroids in a principal coordinates analysis based on the modified Gower distance matrix (Anderson et al., 2006). These data were used in the break point analysis and ordinary least squares regression, described below.

We also used several vegan functions (Oksanen et al., 2013) to determine the response of beta diversity and compositional dissimilarity to variation in spatial and temporal components of experimental fire regimes (number of patches, fire return interval, and burn season). We applied the permustats function to beta diversity results from betadisper to simulate pairwise comparisons among groups. For compositional dissimilarity among groups, we conducted pairwise tests using permutational multivariate analysis of variance (function adonis), and as a further test of whether groups in ordination space are meaningful, we determined whether groups clustered more tightly than randomly expected (function ordiareatest).

#### 2.2.3 Break point analysis

Using the number of patches per landscape as the predictor variable, we used a break point analysis based on piecewise regression to identify break points in three response variables—spatial heterogeneity and temporal variability (as calculated above by random‐effects regression) and beta diversity (as calculated above by multivariate dispersion). Our model treats each number of patches per landscape as a potential single break point in a piecewise regression model and returns the solution with the lowest mean squared error. Break points represent thresholds in the nonlinear association between the response and predictor variables (Toms & Lesperance, 2003); as applied here, the break point identifies the number of patches per landscape that maximizes or minimizes the response variable given the information available in our data.

#### 2.2.4 Ordinary least squares regression

Finally, we fit beta diversity as a response variable against structural heterogeneity and temporal variability as predictor variables in two separate linear regression models validated with the gvlma package (Pena & Slate, 2014). See Appendix S1 for complete script for all analyses.

## 3 RESULTS

### 3.1 Thresholds in heterogeneity, variability, and diversity

**Figure 1:**
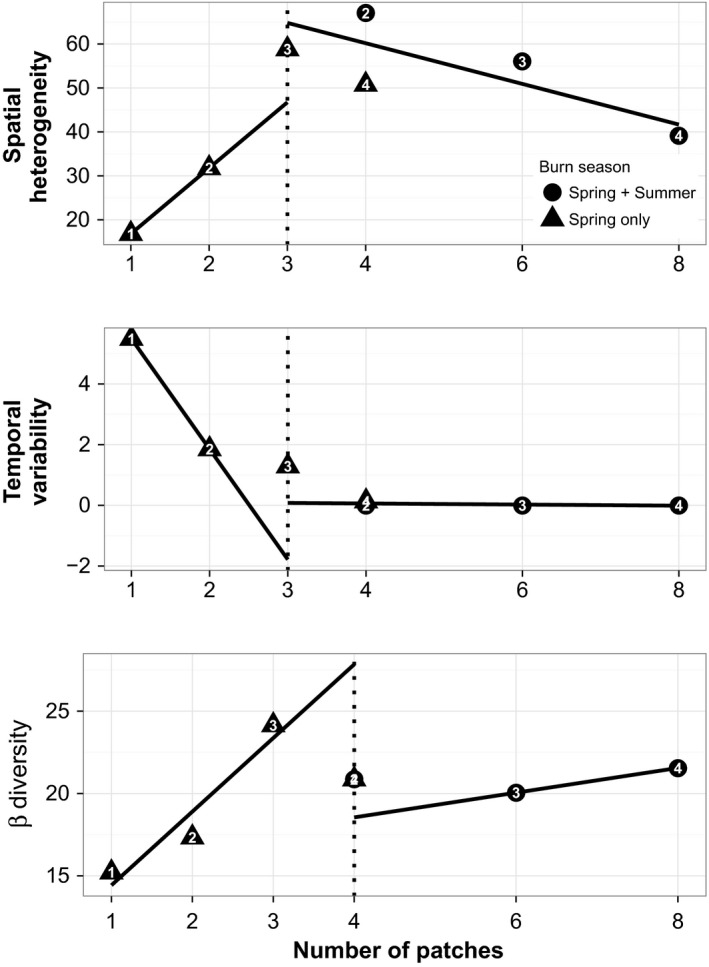
Break points (as broken lines) in linear relationship between three response variables over number of patches per tallgrass prairie landscape, as determined by piecewise regression. Symbol numerals denote fire return interval in years. Spatial heterogeneity and temporal variability measured as variance allocated to plot and year terms in a random‐effects regression model, and beta diversity measured as multivariate dispersion of groups in ordination space (see Methods)

Break point analysis identified break points for spatial heterogeneity and temporal variability at three patches per landscape, representing optimal high and low values, respectively; the beta diversity break point occurred at four patches per landscape (Figure 1). Among the three responses, spatial heterogeneity was unique in having considerable variability among patch number, fire return interval, and season of burn; that is, the greatest amount of heterogeneity occurred in a four‐patch landscape managed with a 2‐year fire return interval achieved through both spring and summer burns, but the other four‐patch landscape—managed with a 4‐year fire return interval and spring‐only fires—had less spatial heterogeneity than both landscapes managed with 3‐year fire return intervals (Figure 1). Notably, this group of four landscapes—the two with 3‐year fire return intervals and the two with four patches—had considerably greater spatial heterogeneity than those managed for one, two, or eight patches (Figure 1).

Temporal variability was an order of magnitude lower than spatial heterogeneity in all landscapes; after declining as patch number increased from one to three, temporal variability was stable and absent at four patches and above (Figure 1), indicating general stability in aboveground biomass in these landscapes with little variability among fire return intervals and burn seasons.

Like spatial heterogeneity, beta diversity increased from one to three patches per landscape and showed little variability among four, six, and eight patches. Unlike spatial heterogeneity, there was greater difference within landscapes with 3‐year fire return intervals: The three‐patch, spring‐only landscape with a 3‐year fire return interval had greater beta diversity than the six‐patch, landscape burned in both spring and summer (Figure 1).

### 3.2 Beta diversity and spatial heterogeneity, temporal variability

**Figure 2:**
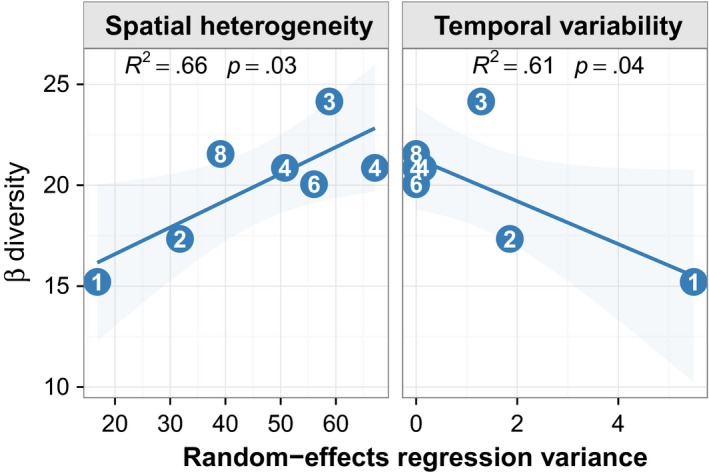
Beta diversity plotted against spatial heterogeneity and temporal variability. Symbol numerals denote number of patches per landscape. Spatial heterogeneity and temporal variability measured as variance allocated to plot and year terms in a random‐effects regression model, and beta diversity measured as multivariate dispersion of groups in ordination space (see Methods)

As expected, there was a positive relationship between beta diversity and spatial heterogeneity (*t*_5_ **= 3.13, *p *=* *.03; *R* ^2^ = .66) (Figure 2). Patterns within the linear trend reflected those of McGranahan et al. (2016) in which the regression line was bound by the single‐patch landscape at the low end and three‐ to four‐patch landscapes on the high end, with six‐ and eight‐patch landscapes occupying the middle. This suggests a diminished effect beyond four patches in the association between beta diversity and spatial heterogeneity (Figure 2).

We also observed the expected negative relationship between beta diversity and temporal variability (*t*_5_ **= –2.77, *p *=* *.04; *R* ^2^ = .61) (Figure 2). Patterns within this relationship were less interpretable because there was little temporal variability in aboveground plant biomass in these landscapes—relative to spatial heterogeneity, temporal variability was an order of magnitude lower—and landscapes with more than three patches had very near zero temporal variability and created a cluster near the *y*‐axis (Figure 2). Spread among the one‐, two‐, and three‐patch landscapes were sufficient, however, to drive a significant, negative relationship and both models passed Pena and Slate's (2014) global test for linear model assumptions.

### 3.3 Beta diversity and compositional dissimilarity

Three‐patch landscapes had the greatest plant functional group beta diversity; significantly greater (*p *<* *.05) than all but the eight‐patch landscape (*p *=* *.15) (Appendix S2). Regarding composition, only one‐ and four‐patch landscapes had significant clusters in ordination space (*p *=* *.001 and *p *=* *.04, respectively). The one‐patch landscape had significantly different composition from all other landscapes (*p *=* *.02) and was characterized by bare ground and graminoid cover, while four‐patch landscapes tended to be characterized by greater forb and litter cover (Appendix S2).

Landscapes with three‐ and 4‐year fire return intervals had the greatest beta diversity, significantly greater than all other landscapes (*p *≤* *.05) and not different from each other (*p *=* *.57). Compositional dissimilarity patterns were consistent with those above because the one‐patch landscape was also managed with a 1‐year fire return interval, and one landscape with a 4‐year fire return interval also had four patches. There was no difference in beta diversity among spring‐only and spring + summer seasonal burn regimes, nor did either group form significantly different‐from‐random clusters in ordination space (Appendix S2).

## 4 DISCUSSION

Across the variables and analyses in this study, a clear pattern emerged: From a heterogeneity‐based management perspective, optimal spatial heterogeneity, temporal variability, and beta diversity occurred at moderate levels of patchiness (three to four patches, or 25%–33% of landscape burned) and three‐ to 4‐year fire return intervals. Additionally, fire return interval and burn season both interacted with patch number such that managers can expect flexibility from managing one or more components of the spatial–temporal fire regime to mitigate constraints, or enhance effects of another component, and still achieve optimal results, especially for spatial heterogeneity.

These results are likely relevant to a breadth of ecosystems worldwide with disturbance regimes characterized by grazing and frequent fire, although direct application might be limited to managed landscapes with fixed areas and control over herbivore numbers such as ranches and smaller conservation areas. While ecological processes in large landscapes such as national parks in southern Africa are driven by interactions between fire and grazing that occur in spatially discrete patches (Archibald et al., 2005), patch number and proportion of the landscape burned are difficult to manage and quantify, and might even be less relevant when animals have access to multiple burned areas and select them not only for forage quality but by other factors such as distance to water and predators. However, fire return interval is still an important management consideration as it balances long‐unburned areas for contrast against maintenance of the grassland state (Archibald et al., 2005; Smith et al., 2013), and prescribed fire use can increase spatial heterogeneity even in large landscapes with natural fire (van Wilgen, Govender, Biggs, Ntsala, & Funda, 2004).

Spatial and temporal components of pyrodiversity can be applied in managed landscapes as well, where spatially patchy fire regimes with livestock grazing increase spatial heterogeneity in vegetation structure and enhance biodiversity conservation (Fuhlendorf et al., 2017). Disturbance‐driven variability in plant biomass and forage quality have been shown in mesic grasslands worldwide (Vignolio et al., 2003; Sensenig, Demment, & Laca, 2010; Allred et al., 2011), and the application of spatially patchy fires in grazed management units creates landscape‐level heterogeneity (McGranahan et al., 2013).

We frame the implications of these results in the context of the U.S. Great Plains, where fire–grazing interactions are widely studied as “pyric herbivory” and applied as “patch burn‐grazing” (Fuhlendorf et al., 2009; McGranahan et al., 2012); however, these points likely apply to mesic grassland worldwide. In our study, four landscapes—the two with 3‐year fire return intervals and the two with four patches—had considerably greater spatial heterogeneity than those managed for one, two, or eight patches, which supports the spatial–temporal fire regime widely applied in the region. While fire–grazing interaction studies are few, those from the Great Plains typically test 3‐year fire return intervals with three patches, and sometimes four and four, respectively, thus burning 25%–33% of a management unit annually (McGranahan et al., 2012; Leis, Morrison, & Debacker, 2013; Larson, Dodds, Whiles, Fulgoni, & Thompson, 2016). More abundant are fire‐only studies, and among those that compare fire return intervals, many support two of our findings here: (1) response is generally low and consistent among 1‐year and 2‐year intervals, and (2) effects appear to peak at three‐ to 4‐year intervals (Veen, Blair, Smith, & Collins, 2008; Chang & Smith, 2013; Ratajczak et al., 2016). Also of interest is how our results and the literature align with reported historical fire return intervals of ca. 3–5 years from the region (Stambaugh, Guyette, Godfrey, McMurray, & Marschall, 2009; Allen & Palmer, 2011).

But consistency between our data and conventions of practice must not be taken as a prescription that three to four patches and three‐ or 4‐year fire return intervals constitute the only, or even the best, management options. Rather, we highlight the flexibility offered by the interactions between patch number and fire frequency, apparent in the range of combinations in the cluster of four landscapes with maximal spatial heterogeneity (Figure 1). Fire managers often start planning with fire return interval because productivity and removal limit seasonal biomass accumulation and fire spread depends on sufficient fuel load and horizontal connectivity (Davies, Svejcar, & Bates, 2009). Constrained by fuelbed conditions, fire regime must support management objectives: Low productivity or high grazing offtake can limit effectiveness of heterogeneity‐based management (McGranahan et al., 2012), and thus, a four‐patch system might facilitate enough accumulation for optimal heterogeneity/stability/diversity outcomes (Figure 1). But higher precipitation or a need to control brush might necessitate shorter intervals (Heisler, Briggs, & Knapp, 2003; Bowles & Jones, 2013), in which case the four‐patch arrangement under a 2‐year fire return interval also achieved objectives of heterogeneity‐based management (Figure 1).

To achieve both a high patchiness (4–6 patches) and a shorter, 2‐year fire return interval, we burned in both spring and summer. Our summer burns were successful, feasible, and had no measurable effect on composition (Appendix S2), which are important considerations in the long and intensifying debate about the traditional practice of spring burning (Engle & Bidwell, 2001; Towne & Craine, 2016). A wider burn window affords managers more opportunity to complete burn operations and potentially avoid dangerous fuel and weather conditions; it also lessens seasonal impacts to air quality and might stabilize plant community diversity because when species differ seasonally in their sensitivity to fire, variability might equalize severity over time (Weir, 2011; Pavlovic, Leicht‐Young, & Grundel, 2011; Towne & Craine, 2016). That we saw no difference among spring and summer burns indicates managers can consider summer fire without compromising heterogeneity‐based objectives. And of practical note for livestock production objectives, cattle weights are not reduced on rangelands managed for heterogeneity, and in fact, spatial heterogeneity appears to stabilize cattle weight during drought (Allred, Scasta, Hovick, Fuhlendorf, & Hamilton, 2014).

In a broader context, if there is any prescription in our results it should be to question how ecologists worldwide study spatial–temporal components of fire regime and balance tradeoffs between replication and regression. The conventional approach that compares replicated management practices—often a single heterogeneity‐based regime against a homogeneously managed “control”—derives from a tradition of means comparison and statistical, rather than biological, interactions (Fuhlendorf et al., 2017) that is criticized for an inability to identify mechanisms or test for nonlinear effects (Foster et al., 2016). Our study offers a direct response in that we (1) experimentally created a gradient of patchiness and (2) specifically applied a regression approach sensitive to nonlinear relationships. But creating a broad gradient in patch number required manipulating fire return intervals and burn seasons such that no combination of the three variables was replicated. Depending on one's perspective, this either contributes to ecology's alleged replication crisis or serves as an example of a large, unreplicated experiment that tests a logistically challenging hypothesis along an ecological gradient (Barley & Meeuwig, 2017). To balance replication with regression, we must (1) accept that complete‐block designs inclusive of all combinations are impossible (and unnecessary when biologically illogical combinations are discarded), and (2) distinguish important variables or combinations from those already understood.

Meta‐analysis suggests patch contrast explains why some applications of spatially discrete disturbance beget spatially heterogeneous structure and others do not (McGranahan et al., 2012), while recent advances in ecological theory explain how within‐patch and between‐patch dynamics create contrast and enhance landscape‐level heterogeneity and stability (Wang & Loreau, 2014; McGranahan et al., 2016). Meanwhile, we demonstrate here that spatial heterogeneity and temporal stability are associated with beta diversity in standard community composition data (Figure 2), making it possible for researchers without the data, or capacity to apply complicated models, to still make inference into heterogeneity‐related ecosystem functions. Ecologists must apply these theories and develop robust experiments that focus on relevant increments along patchiness and disturbance gradients and replicate the understudied combinations of fire seasonality, behavior, and effects.

## CONFLICT OF INTEREST

The authors declare no conflict of interests.

## AUTHOR CONTRIBUTIONS

D.A.M. conceived the ideas for the paper, designed and conducted analysis, and led the writing of the manuscript. T.J.H. collected the data in the initial study and contributed to writing. R.D.E. contributed to the conception and leadership of the original study. D.M.E. contributed to the conceptual development of the present paper. S.D.F. contributed to the conception and leadership of the original study and conceptual development of the present paper. All authors contributed to writing the present paper and gave final approval for submission.
