# Supplementary material for: Moderate patchiness optimizes heterogeneity, stability, and beta diversity in mesic grassland
Source: Ecol Evol. 2018 Apr 20;8(10):5008–15. doi: 10.1002/ece3.4081 (PMC5980247; doi:10.1002/ece3.4081)
Supplement: Supplementary file 1 [file ECE3-8-5008-s001.pdf]

Appendix S1 - Supplemental information on methods

Experimental design

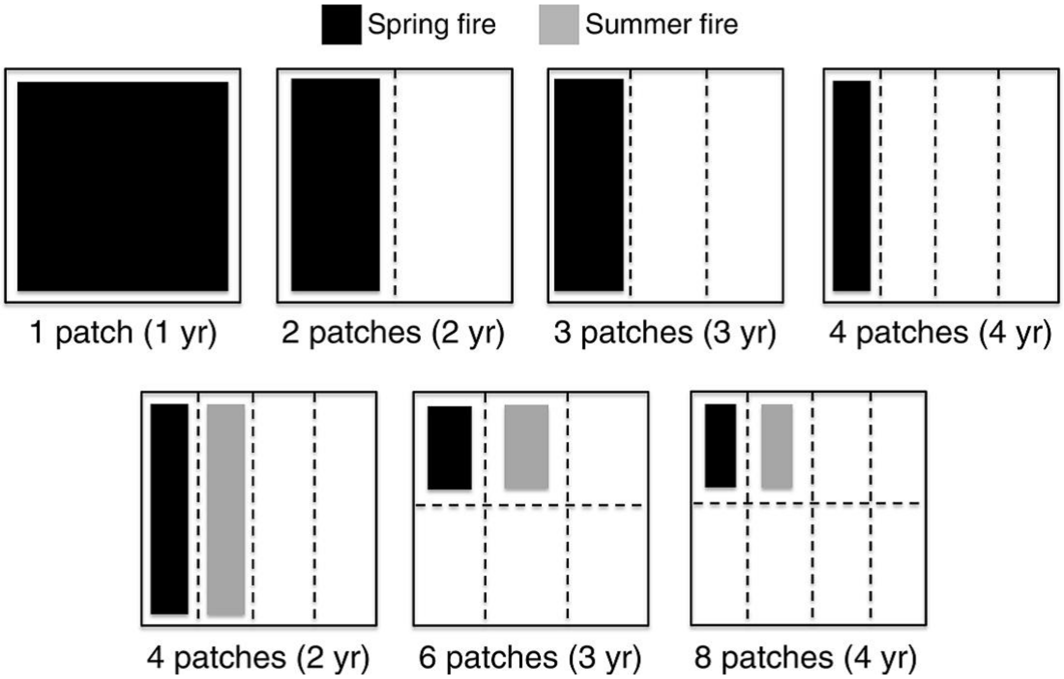

Figure 1: Schematic of experimental design. Each box represents an exterior fence of an experimental landscape, below which is indicated the number of patches within each landscape and the fire return interval in years (parenthetically). Dashed lines denote patches created by discrete fire. Dark boxes represent spring fires (March–April) and gray boxes represent summer fires (late July–August). Experimental landscapes ranged from 430 to 980 ha (mean = 627 ha) and patch sizes ranged from 79 to 378 ha (mean = 170 ha). From Hovick et al. 2015. Spatial heterogeneity increases diversity and stability in grassland bird communities. Ecological Applications 25:662–672.

Table 1: Summary of experimental pastures and spatial and temporal aspects of fire management during study.

Landscape name	Number of patches	Fire return interval	Burn season(s)
Greeley	1	1	Spring only
East Stish	2	2	Spring only
Mary L.	3	3	Spring only
West Stish	4	2	Spring + Summer
Sand Creek	4	4	Spring only
West John Lee	6	3	Spring + Summer
East John Lee	8	4	Spring + Summer

R script

Custom functions

`RE.gam.var`

A variance partitioning method based on random-effects generalized linear regression model using `glmer` with `Gamma(link = "identity")` to calculate variance from spatial and temporal terms in visual obstruction data.

```
> RE.gam.var <- function(x) {
+   var.mod <- glmer(value~(1|year)+ (1|transect.num:year),
+                    Gamma(link = "identity"), data=x)
+   transect.var <- (attr(VarCorr(var.mod)$'transect.num:year',"stddev"))^2
+   year.var <- (attr(VarCorr(var.mod)$'year',"stddev"))^2
+   var.results<-array(NA,c(1,2))
+   colnames(var.results)<-c("transect.var","year.var")
+   var.results[1,] <- round(c(transect.var, year.var), 4)
+   return(var.results)        }
```

`patch.PWR`

Performs piecewise regression by determining the breakpoint with the lowest mean squared error (MSE) and extracting regression coefficients for fitting trendlines to data on either side of the breakpoint.

```
> patch.PWR <- function(dat) {
+   x <- dat$patches
+   y <- dat$value
+
+   breaks <- x[which(x >= 1 & x <= 8)]
+   mse <- numeric(length(breaks))
+   for(i in 1:length(breaks)){
+     piecewise1 <- lm(y ~ x*(x < breaks[i]) +
+                        x*(x>=breaks[i]))
+     mse[i] <- summary(piecewise1)[6]}
+   mse <- as.numeric(mse)
+   bp <- breaks[which(mse==min(mse))][[1]]
+   piecewise2 <- lm(y ~ x*(x < bp) +
+                      x*(x > bp))
+    pw.coefs <- round(piecewise2$coefficients, 3)
+      pdf(file=NULL)
+     if (is.na(pw.coefs[[2]] + pw.coefs[[5]]))
+       {p1 <- data.frame(x=NA, y=NA)} else
+     {p1 <- curve((pw.coefs[[1]] + pw.coefs[[3]]) +
+                        (pw.coefs[[2]] + pw.coefs[[5]])*x,
+                 add=FALSE, from=1, to=bp) }
+      if (is.na(pw.coefs[[1]] + pw.coefs[[4]]))
+      {p2 <- data.frame(x=NA, y=NA)} else
+       { p2 <- curve((pw.coefs[[1]] + pw.coefs[[4]]) +
+                   pw.coefs[[2]]*x, add=FALSE, from=bp, to=max(x)) }
+     dev.off()
+ pw.results <- data.frame(bp=bp,# r2=round(summary(piecewise2)$r.squared,2),
+                          p1.x1=p1$x[1], p1.x2=p1$x[length(p1$x)],
+                          p1.y1=p1$y[1], p1.y2=p1$y[length(p1$y)],
+                          p2.x1=p2$x[1], p2.x2=p2$x[length(p2$x)],
+                          p2.y1=p2$y[1], p2.y2=p2$y[length(p2$y)])
+ return(pw.results)}
```

pairwise.adonis

Pairwise comparison of composition among two or more grouping factors in a multivariate dataset using the vegan function adonis.

```
> pairwise.adonis <-function(x,factors, sim.method, p.adjust.m){
+   require(vegan)
+     co = as.matrix(combn(unique(factors),2))
+     pairs = c()
+     F.Model =c()
+     R2 = c()
+     p.value = c()
+
+   for(elem in 1:ncol(co)){
+     ad = adonis(x[factors %in% c(as.character(co[1,elem]),
+                                  as.character(co[2,elem])),] ~
+                    factors[factors %in% c(as.character(co[1,elem]),
+                                           as.character(co[2,elem]))] ,
+                  method =sim.method);
+     pairs = c(pairs,paste(co[1,elem],'-',co[2,elem]));
+     F.Model = round(c(F.Model,ad$aov.tab[1,4]),2);
+     R2 = round(c(R2,ad$aov.tab[1,5]),2);
+     p.value = round(c(p.value,ad$aov.tab[1,6]),2)
+           }
+   p.adj = round(p.adjust(p.value,method=p.adjust.m),2)
+   pairw.res = data.frame(pairs,F.Model,p.adj)
+   return(pairw.res) }
```

A complete R session

The following script has been fully compiled with R + Sweave.

```
[1] "At this point workspace should contain:"

[1] "tgpp.med" "tgpp.vor"

  patches landscape transect.num year graminoid forb shrub litter bare vor
1       8       EJL            1    1        63   16     0     16   86  18
2       2        ES            1    1        63   38     0     16   63  44
3       1        GR            1    1        63   38     0     38   38  49
4       3        ML            1    1        63   38     0     63   38  55
5       4        SC            1    1        86   38     0     98    0  52
6       6       WJL            1    1        86   16     3     98    0  71
  depth fri season
1     0   4  Sp+Su
2     0   2     Sp
3     0   1     Sp
4     0   3     Sp
5     3   4     Sp
6    15   3  Sp+Su
```

Ordination analysis

```
> tgpp.dist <- vegdist(tgpp.med[5:9], method="altGower")
> pcoa <- capscale(tgpp.med[5:9] ~ 1, method="altGower",
+                  sqrt.dist = FALSE)
> pcoa.scores <- scores(pcoa, scaling=0)
> pcoa.spp <- data.frame(pcoa.scores$species)
> pcoa.spp$species <- rownames(pcoa.spp)
```

```
> pcoa.sites <- data.frame(tgpp.med[c(1:2,10)],
+                 PCoA1.score=round(pcoa.scores$sites[,1],3),
+                 PCoA2.score=round(pcoa.scores$sites[,2], 3) )
>   pcoa.sites$fri <- revalue(pcoa.sites$landscape,
+                                     c("GR"="1", "ES"="2", "ML"="3", "EJL"="4",
+                                       "SC"="4", "WS"="2", "WJL"="3"))
>   pcoa.sites$season <- revalue(pcoa.sites$landscape,
+                                       c("GR"="Sp", "ES"="Sp", "ML"="Sp",
+                                         "EJL"="Sp+Su", "SC"="Sp",
+                                         "WS"="Sp+Su", "WJL"="Sp+Su"))
> #
> # Patch number analysis
> # beta diversity
> patches.bd <- betadisper(tgpp.dist, factor(tgpp.med$patches),
+                          type="centroid")
> patches.pm <- permutest(patches.bd,
+                              permutations=99, pairwise=TRUE)
> patches.ps <- permustats(patches.pm)
> patches.dtc <- data.frame(distance=patches.bd$distances,
+                 group=patches.bd$group)
> # Dissimilarity
> patches.ad <- pairwise.adonis(x=tgpp.med[5:9], factors=tgpp.med$patches,
+               sim.method="altGower", p.adjust.m ='bonferroni')
>   # ordiareatest(pcoa, pcoa.sites$patches, kind="se")
> #
> # Fire Return Interval
> # beta diversity
> fri.bd <- betadisper(tgpp.dist, factor(tgpp.med$fri),
+                          type="centroid")
> fri.pm <- permutest(fri.bd, permutations=99, pairwise=TRUE)
> fri.ps <- permustats(fri.pm)
> fri.dtc <- data.frame(distance=fri.bd$distances,
+                       group=(as.numeric(as.character(fri.bd$group))))
> # dissimilarity
> fri.ad <- pairwise.adonis(x=tgpp.med[5:9], factors=tgpp.med$fri,
+               sim.method="altGower", p.adjust.m ='bonferroni')
>  # ordiareatest(pcoa, pcoa.sites$fri, kind="se")
> #
> # Burn season
> # beta diversity
> seas.bd <- betadisper(tgpp.dist, factor(tgpp.med$season),
+                          type="centroid")
> seas.pm <- permutest(seas.bd, permutations=99, pairwise=TRUE)
> seas.ps <- permustats(seas.pm)
> seas.dtc <- data.frame(distance=seas.bd$distances,
+                 group=seas.bd$group)
> # Dissimilarity
> seas.ad <- pairwise.adonis(x=tgpp.med[5:9], factors=tgpp.med$season,
+               sim.method="altGower", p.adjust.m ='bonferroni')
>   # ordiareatest(pcoa, pcoa.sites$season, kind="se")
```

Random-effects regression

```
> vor.var <- ddply(.data=tgpp.vor,
+                       .(patches, landscape),
+                       .fun=RE.gam.var)
> # colnames(vor.var)[3:4] <- c("raw.spatial","raw.temporal")
```

```
> md <- merge(x=vor.var, y=ddply(patches.dtc, .(group),
+                          summarise, ord.dist=round(mean(distance),2)),
+             by.x="patches", by.y="group")
> #
> var.dat <- cbind(md[1:2], stack(md[3:5]))
> colnames(var.dat)[3:4] <- c("value", "variable")
> var.dat$fri <- revalue(var.dat$landscape,
+                                    c("GR"="1", "ES"="2", "ML"="3", "EJL"="4",
+                                      "SC"="4", "WS"="2", "WJL"="3"))
> var.dat$season <- revalue(var.dat$landscape,
+                          c("GR"="Sp", "ES"="Sp", "ML"="Sp",
+                            "EJL"="Sp+Su", "SC"="Sp",
+                            "WS"="Sp+Su", "WJL"="Sp+Su"))
> var.dat$label <- revalue(var.dat$variable,
+                                  c("transect.var"="Spatial heterogeneity",
+                                    "year.var"="Temporal variability",
+                                   "ord.dist"="beta~diversity"))
```

Piecewise regression

```
> pwr.coords <- ddply(var.dat, .(label, variable),
+                     .fun=patch.PWR)
```

Testing beta diversity against heterogeneity, stability

```
> tv.m <- lm(log(ord.dist+1) ~ transect.var, md)
> tv.m2 <- gvlma(tv.m)
> #
> yv.m <- lm(log(ord.dist+1) ~ year.var, md)
> yv.m2 <- gvlma(yv.m)
```

```
> sessionInfo();

R version 3.3.3 (2017-03-06)
Platform: x86_64-w64-mingw32/x64 (64-bit)
Running under: Windows 10 x64 (build 14393)

locale:
[1] LC_COLLATE=English_United States.1252
[2] LC_CTYPE=English_United States.1252
[3] LC_MONETARY=English_United States.1252
[4] LC_NUMERIC=C
[5] LC_TIME=English_United States.1252

attached base packages:
[1] stats     graphics  grDevices utils     datasets  methods   base

other attached packages:
[1] vegan_2.4-2     lattice_0.20-34 permute_0.9-4   gvlma_1.0.0.2
[5] gridExtra_2.2.1 ggplot2_2.2.1   plyr_1.8.4      lme4_1.1-12
[9] Matrix_1.2-8

loaded via a namespace (and not attached):
 [1] Rcpp_0.12.9      cluster_2.0.5    splines_3.3.3    MASS_7.3-45
 [5] munsell_0.4.3    colorspace_1.3-2 minqa_1.2.4      tools_3.3.3
 [9] parallel_3.3.3   grid_3.3.3       gtable_0.2.0     nlme_3.1-131
[13] mgcv_1.8-17      pacman_0.4.1     lazyeval_0.2.0   assertthat_0.1
[17] tibble_1.2       nloptr_1.0.4     scales_0.4.1
```
