# Supplementary material for: Moderate patchiness optimizes heterogeneity, stability, and beta diversity in mesic grassland
Source: Ecol Evol. 2018 Apr 20;8(10):5008–15. doi: 10.1002/ece3.4081 (PMC5980247; doi:10.1002/ece3.4081)
Supplement: Supplementary file 2 [file ECE3-8-5008-s002.pdf]

Appendix S2 - Supplemental results

β diversity and compositional dissimilarity across grouping factors

Number of patches

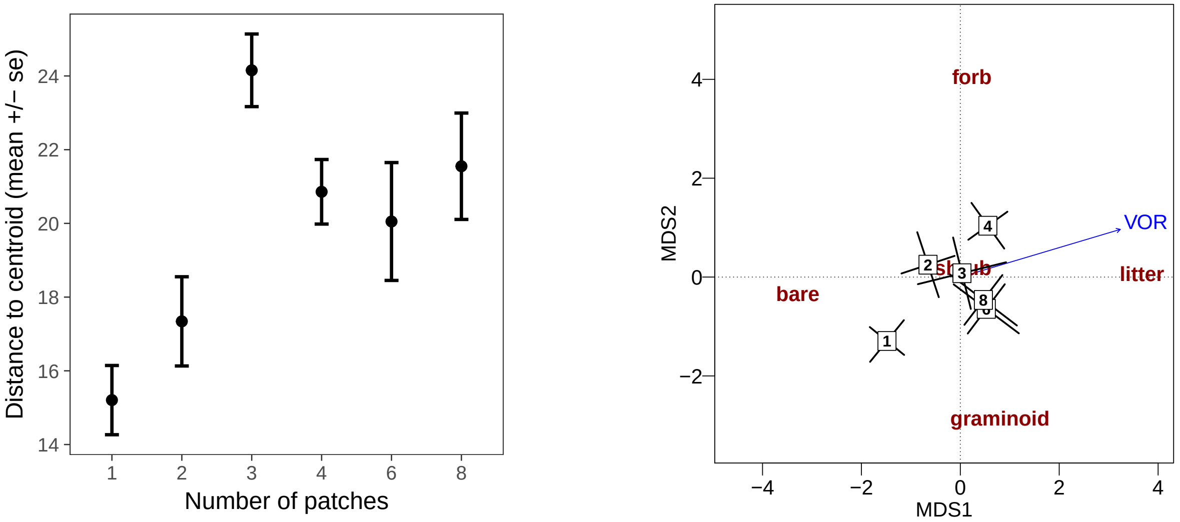

(a) β diversity measured as mean distance to centroid ($\pm$ standard error) by number of patches/landscape using `betadisper` function.

(b) Group centroids ($\pm$ se) by number patches/landscape in Principal Coordinates Analysis using `altGower` distance. *Blue VOR arrow* indicates visual obstruction gradient, *bold brown labels* indicates species scores. The 1-patch and 4-patch landscapes had significant clusters (P=0.001 & P=0.04, respectively).

Figure 1: Graphic representations of (a) β diversity and (b) compositional dissimilarity by number of patches/landscape. See Table 1 for pairwise comparisons using permutation tests in `vegan`.

Table 1: Pairwise comparisons of β diversity and compositional dissimilarity across number of patches per landscape, as measured by `vegan` objects. Blue boxes highlight significant differences in permutation tests at $\alpha = 0.05$.

	test.statistic	P
Overall (F)	6.39	0.01
1-2 (t)	-1.39	0.19
1-3 (t)	-6.58	0.01
1-4 (t)	-4.02	0.01
1-6 (t)	-2.61	0.01
1-8 (t)	-3.68	0.01
2-3 (t)	-4.34	0.01
2-4 (t)	-2.33	0.09
2-6 (t)	-1.35	0.21
2-8 (t)	-2.23	0.11
3-4 (t)	2.28	0.01
3-6 (t)	2.15	0.03
3-8 (t)	1.47	0.15
4-6 (t)	0.48	0.77
4-8 (t)	-0.43	0.73
6-8 (t)	-0.70	0.49

(a) Pairwise comparisons of β diversity (mean distance to centroid) of groups as calculated by call to `permustats` on `betadisper` object in `vegan`.

pairs	F.Model	P.adj
8 - 2	6.19	0.19
8 - 1	17.01	0.02
8 - 3	1.35	1
8 - 4	4.02	0.34
8 - 6	0.02	1
2 - 1	9.08	0.02
2 - 3	3.17	0.69
2 - 4	9.19	0.03
2 - 6	7.43	0.04
1 - 3	10.82	0.02
1 - 4	34.50	0.02
1 - 6	17.25	0.02
3 - 4	3.70	0.55
3 - 6	1.77	1
4 - 6	5.29	0.18

(b) Pairwise comparisons of group-level compositional dissimilarity as calculated by call to `pairwise.adonis`.

Fire return interval

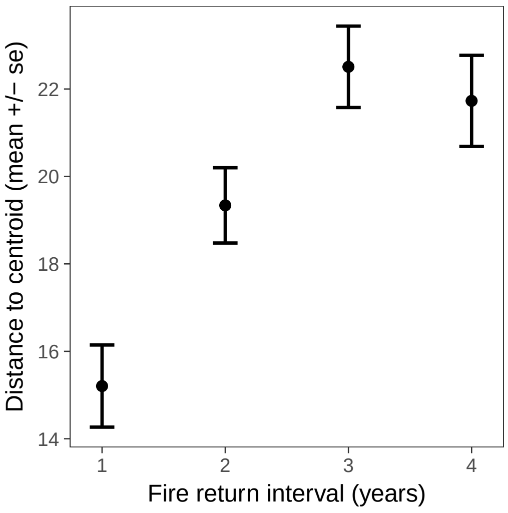

(a) β diversity measured as mean distance to centroid ($\pm$ standard error) by fire return interval using `betadisper` function.

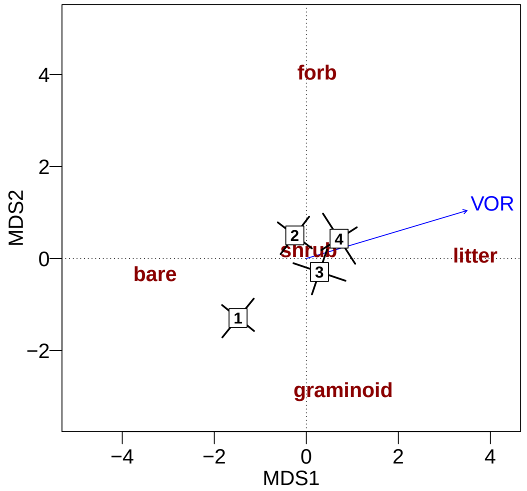

(b) Group centroids ($\pm$ se) by fire return interval in PCoA using `altGower` distance. *Blue VOR arrow* indicates visual obstruction gradient, *bold brown labels* indicates species scores. The 1-year FRI landscape had a significant cluster (P=0.001); the 2-year FRI cluster was marginally significant(P=0.08).

Figure 2: Graphic representations of (a) β diversity and (b) compositional dissimilarity by fire return interval. See Table 2 for pairwise comparisons using permutation tests in `vegan`.

Table 2: Pairwise comparisons of β diversity and compositional dissimilarity across fire return intervals, as measured by `vegan` objects. Blue boxes highlight significant differences in permutation tests at $\alpha = 0.05$.

	test.statistic	P
Overall (F)	8.34	0.01
4-2 (t)	1.77	0.05
4-1 (t)	4.03	0.01
4-3 (t)	-0.56	0.57
2-1 (t)	2.98	0.01
2-3 (t)	-2.50	0.03
1-3 (t)	-4.99	0.01

(a) Pairwise comparisons of β diversity (mean distance to centroid) of groups as calculated by call to `permustats` on `betadisper` object in `vegan`.

pairs	F.Model	P.adj
4 - 2	6.24	0.03
4 - 1	29.21	0.01
4 - 3	2.70	0.42
2 - 1	17.48	0.01
2 - 3	4.55	0.14
1 - 3	15.66	0.01

(b) Pairwise comparisons of group-level compositional dissimilarity as calculated by call to `pairwise.adonis`; P adjusted by Bonferroni.

Season of burn

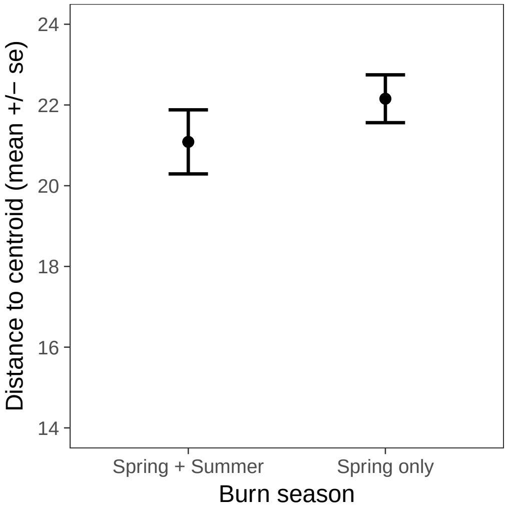

(a) β diversity measured as mean distance to centroid ($\pm$ standard error) by burn season using `betadisper` function.

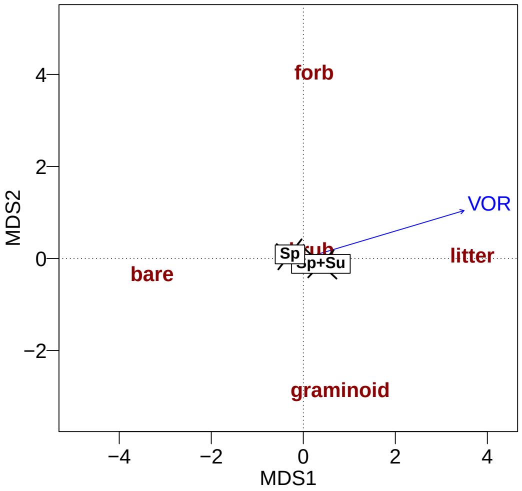

(b) Centroids ($\pm$ se) of groups defined by season of burn (Spring only or Spring + Summer). *Blue VOR arrow* indicates visual obstruction gradient, *bold brown labels* indicates species scores. Neither cluster was significantly tighter than random.

Figure 3: Graphic representations of (a) β diversity and (b) compositional dissimilarity by burn season regime. See Table 3 for pairwise comparisons using permutation tests in `vegan`.

Table 3: Pairwise comparisons of β diversity and compositional dissimilarity across two burn season regimes, as measured by `vegan` objects. Blue boxes highlight significant differences in permutation tests at $\alpha = 0.05$.

	test.statistic	P
Overall (F)	1.21	0.24
Sp+Su-Sp (t)	-1.10	0.31

(a) Pairwise comparisons of β diversity (mean distance to centroid) of groups as calculated by call to `permustats` on `betadisper` object in `vegan`.

pairs	F.Model	P.adj
Sp+Su - Sp	5.65	0.01

(b) Pairwise comparisons of group-level compositional dissimilarity as calculated by call to `pairwise.adonis`; P adjusted by Bonferroni.
